# Durable complete radiographic and biochemical response to olaparib in BRCA2-mutated metastatic castration-resistant prostate cancer: a long-term responder case

**DOI:** 10.1016/j.eucr.2026.103356

**Published:** 2026-01-27

**Authors:** Fumihiro Ito, Koki Kobayashi, Gaku Hayashi, Shunsuke Kamijo, Takashi Fujita

**Affiliations:** Department of Urology, Gifu Prefectural Tajimi Hospital, Tajimi, Japan

**Keywords:** Bone scan index, BRCA2 mutation, Complete response, mCRPC, Olaparib

## Abstract

Poly(ADP-ribose) polymerase inhibitors are an important option for metastatic castration-resistant prostate cancer (mCRPC) with homologous recombination repair gene alterations, although durable complete responses remain rare. We report a 72-year-old man with BRCA2-mutated mCRPC who achieved a sustained complete response to olaparib monotherapy. After progression on androgen deprivation therapy, abiraterone, enzalutamide, and docetaxel, genomic profiling identified a pathogenic BRCA2 alteration. Olaparib induced an undetectable prostate-specific antigen within three months, maintained for over 27 months, with complete resolution of bone metastases. This case highlights the potential for durable responses to olaparib and the importance of early genomic testing.

## Highlights

• BRCA2-mutated mCRPC achieved durable complete response with olaparib monotherapy
• Comprehensive genomic profiling identifies candidates for PARP inhibitor therapy
• Bone scan index objectively confirmed deep radiographic response
• Selected patients may achieve long-term benefit without cumulative toxicity

## 1 Introduction

Poly(ADP-ribose) polymerase (PARP) inhibitors are an established therapy for metastatic castration-resistant prostate cancer (mCRPC) with homologous recombination repair (HRR) gene alterations, particularly BRCA2 mutations. The PROfound trial demonstrated improved survival with olaparib in this setting, although complete and sustained responses remain rare.^1^^,^^2^ We report a Japanese patient who achieved a durable complete remission exceeding two years with olaparib monotherapy, underscoring the potential for profound benefit in selected individuals.

## 2 Case presentation

**Fig. 1:**
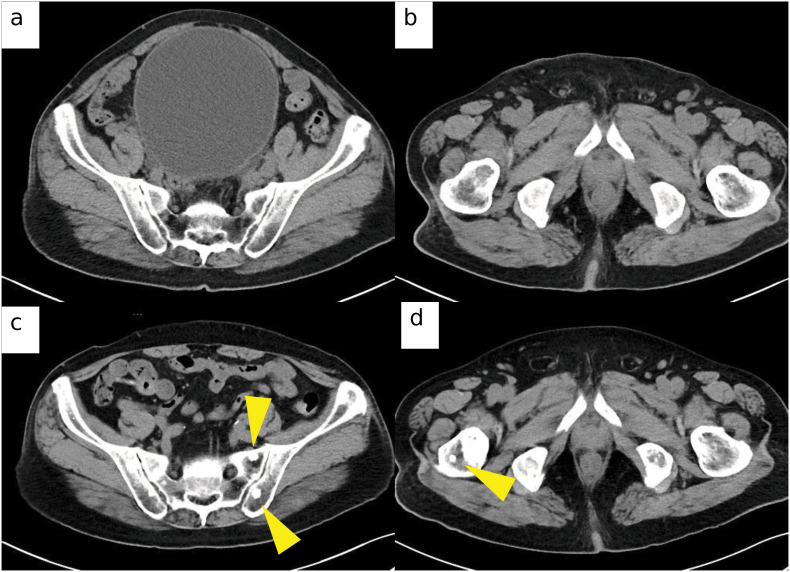
**CT images demonstrating disease course prior to olaparib initiation.** (a) Axial CT image at initial diagnosis demonstrating osteoblastic metastasis in the pelvic bone. (b) Axial CT image at initial diagnosis showing osteoblastic metastasis in the femur. (c) Axial CT image obtained at prostate-specific antigen (PSA) progression during enzalutamide therapy, demonstrating increased osteoblastic lesions consistent with radiologic progression. (d) Axial CT image at PSA progression during enzalutamide therapy confirming further progression of sclerotic bone metastases. All images are axial CT scans acquired with a 5-mm slice thickness.

A 72-year-old man presented with dysuria and was diagnosed with prostate adenocarcinoma involving all 12 biopsy cores (Gleason 4 + 5 = 9). Imaging demonstrated multiple osteoblastic bone metastases (cT4N1M1b), and prostate-specific antigen (PSA) was 27 ng/mL. Baseline CT images are shown in Fig. 1a and b. He had no family history of prostate or breast/ovarian cancer.

Androgen deprivation therapy (ADT) with goserelin plus abiraterone (1000 mg/day) and prednisolone (5 mg/day) initially reduced PSA, but biochemical and radiographic progression occurred after approximately eight months (PSA 6.0 ng/mL). Enzalutamide was then initiated, and further progression occurred within three months (PSA 31.2 ng/mL), CT showing increased sclerotic lesions (Fig. 1c and d). Although bone scintigraphy was not performed, mCRPC was diagnosed based on PSA rise and imaging, consistent with Prostate Cancer Clinical Trials Working Group 3 criteria.^3^ Disease progressed after docetaxel. Docetaxel (75 mg/m2 every 3 weeks) was initiated but discontinued after the first cycle due to biochemical non-response and treatment intolerance. PSA increased from 31.2 ng/mL before initiation to 54.9 ng/mL at early reassessment.

Comprehensive genomic profiling of the tumor was performed using the FoundationOne CDx DX2 assay (tumor-only). The analysis identified a pathogenic BRCA2 rearrangement involving intron 24. Co-existing somatic alterations included ACVR1B deletion (intron 1–exon 3), JAK2 L545_F547del, RB1 splice-site deletion (1420_1421 + 30del32), and SETD2 E1043∗. No additional pathogenic variants were detected in other homologous recombination repair genes such as ATM, CHEK2, PALB2, RAD51 family, or CDK12.

This profiling was performed using a tumor-only assay; therefore, the germline or somatic origin of the BRCA2 rearrangement could not be determined. Copy-number or allele-specific analyses were not available, and thus bi-allelic inactivation of BRCA2 could not be confirmed.

**Fig. 2:**
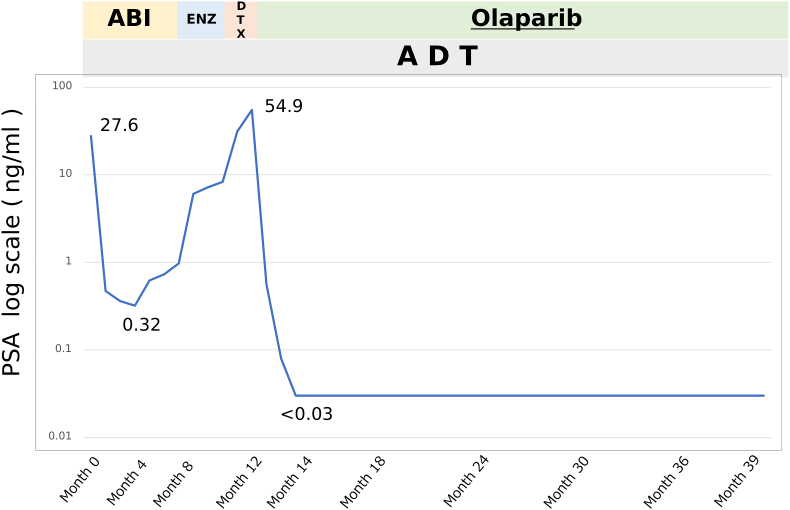
**Clinical course and PSA kinetics during sequential systemic therapy**. PSA levels (log scale) during androgen deprivation therapy (ADT), abiraterone (ABI), enzalutamide (ENZ), and a short course of docetaxel (DTX), followed by olaparib. Rising PSA during sequential androgen receptor–targeted therapy and docetaxel prompted transition to olaparib based on the BRCA2 alteration, resulting in rapid and durable biochemical remission (>27 months). Docetaxel was discontinued after one cycle due to biochemical non-response and intolerance. Timeline Month 0: ADT + abiraterone initiated Month 8: Switched to enzalutamide for PSA progression Month 11: mCRPC confirmed based on rising PSA and CT progression Docetaxel (1 cycle) started Month 12: Olaparib initiated Month 39: Last follow-up; PSA undetectable Note: PSA plotted on logarithmic scale.

Given the presence of a deleterious BRCA2 alteration, olaparib (600 mg/day) was initiated. The subsequent clinical course and PSA kinetics during sequential systemic therapies are shown in Fig. 2.

## 3 Discussion

This case illustrates an exceptional and durable complete response to olaparib monotherapy in BRCA2-mutated mCRPC, with sustained biochemical and radiographic remission for 27 months and no treatment-related toxicity. Such responses remain extremely uncommon, indicating an “ultra-responder” phenotype.

### 3.1 Long-term efficacy of PARP inhibition

In the PROfound trial, median radiographic progression-free survival for BRCA1/2- or ATM-mutated mCRPC treated with olaparib was 7.4 months, and complete responses were rare.^1^ Subsequent phase 3 trials of PARP inhibitor combinations, including PROpel, MAGNITUDE, and TALAPRO-2, demonstrated improved outcomes but still infrequent durable complete remission.^4^^,^^5^ Our patient's 27-month complete response markedly exceeds typical outcomes, supporting the existence of a small subset of patients with exceptional PARP inhibitor sensitivity.^6^^,^^7^

### 3.2 Imaging and diagnostic considerations

**Fig. 3:**
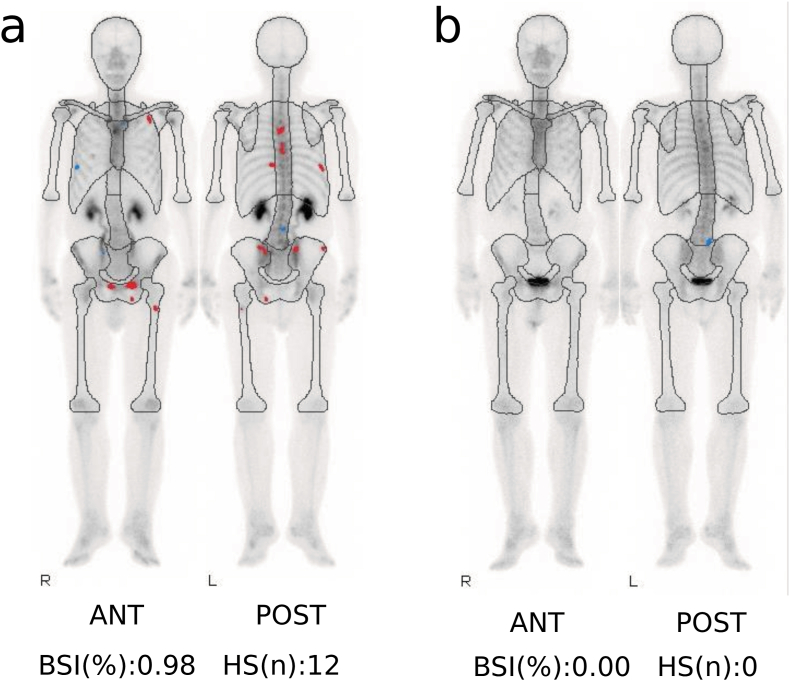
**Bone scintigraphy before and after olaparib therapy**. (a) Baseline bone scan showing multiple osteoblastic metastases (bone scan index [BSI] 0.98). (b) Follow-up scan after 27 months of olaparib demonstrating complete resolution of metastatic lesions (BSI 0.00). Images were acquired using a dual-head SPECT gamma camera, and BSI was quantified via automated software analysis.

At metastatic castration-resistant disease conversion, bone scintigraphy was not repeated because disease progression was already clearly supported by PSA kinetics and CT findings, and immediate treatment modification was prioritized in accordance with institutional clinical practice. Prostate Cancer Clinical Trials Working Group 3 criteria allow diagnosis based on PSA progression and radiographic findings without mandatory bone scintigraphy.^3^ In practice, real-world constraints may necessitate reliance on PSA kinetics and CT. However, when feasible, baseline and periodic bone scintigraphy remain ideal for standardized evaluation. In this case, subsequent bone scintigraphy demonstrated complete resolution of lesions, with a BSI of 0.00, providing objective quantitative confirmation of remission (Fig. 3). The BSI and automated BSI have been validated as reproducible imaging biomarkers correlated with survival and treatment response in mCRPC.^8^^,^^9^ Interpretation of bone lesions during therapy can be challenging, as osteoblastic changes may reflect either progression or flare. Here, rising PSA and radiographic changes supported progression, prompting timely treatment modification. The subsequent profound response underscores appropriate clinical judgment and highlights the role of quantitative imaging metrics in confirming therapeutic effect. BSI 0.00 served as an objective, quantitative confirmation of complete remission, mitigating concerns regarding flare phenomena or inter-reader variability.

Although only one cycle of docetaxel was administered, early biochemical non-response and intolerance justified discontinuation. We acknowledge that limited taxane exposure may theoretically influence subsequent treatment responsiveness; however, the absence of PSA decline and poor tolerability supported early transition to genomically guided therapy in this patient. Importantly, this does not

Diminish the clinical significance of the profound response to olaparib; rather, it highlights the value of genomic-guided treatment selection when conventional therapy is neither tolerable nor effective. This sequence underscores how timely transition to PARP inhibition, prompted by genomic profiling, can lead to dramatic and durable disease control in selected patients.

### 3.3 Biological basis of the exceptional response

The exceptional depth and durability of response observed in this case may be explained by the underlying genomic profile. The tumor harbored a pathogenic BRCA2 rearrangement, a canonical driver of homologous recombination deficiency and a well-established predictor of profound sensitivity to PARP inhibition (10,11). Although bi-allelic BRCA2 inactivation could not be formally confirmed because copy-number and LOH data were unavailable, the presence of a structural rearrangement in intron 24 strongly suggests functional disruption of BRCA2. Nevertheless, because this analysis was performed using a tumor-only assay without copy-number or allele-specific assessment, biallelic BRCA2 inactivation cannot be definitively confirmed. Therefore, this case supports—but does not prove—the presence of a functionally homologous recombination–deficient phenotype. Tumor-only genomic assays may overestimate actionable HRD status, and careful interpretation is warranted when inferring therapeutic sensitivity from single-hit alterations.

In addition, co-existing alterations in RB1 and SETD2 may contribute to an “ultra-responder” phenotype. SETD2 loss impairs DNA-damage signaling and chromatin remodeling, enhancing vulnerability to PARP inhibition (11). RB1 inactivation has been associated with increased genomic instability and a more

HRD-like phenotype in advanced prostate cancer (10,11), biological contexts that amplify reliance on PARP-mediated DNA repair. The absence of competent resistance-driving pathways and the lack of prior platinum therapy may further explain the unusually durable remission lasting more than 27 months.

### 3.4 Comparison with prior reports

Only isolated reports describe sustained complete responses beyond two years. Ma et al. reported germline BRCA2-mutated mCRPC with complete remission exceeding 24 months,^6^ and Hirayama et al. described a similar long-term response in a Japanese patient guided by genomic profiling.^7^ These cases, together with ours, support the existence of an “ultra-responder” subgroup with preserved apoptotic pathways and limited intratumoral heterogeneity.

### 3.5 Clinical implications and genomic testing

Current clinical guidelines recommend routine testing for HRR gene alterations in advanced prostate cancer.^10^^,^^11^ Early genomic profiling enabled timely initiation of olaparib in this case, avoiding further ineffective androgen-signaling therapy or chemotherapy. Although combination approaches may offer additive benefit in some settings,^4^^,^^5^ this case demonstrates that monotherapy can yield durable, toxicity-free disease control when a strong homologous recombination deficiency driver mutation is present.

### 3.6 Resistance and surveillance

Acquired resistance to PARP inhibition may arise through BRCA2 reversion mutations or replication fork stabilization.^12^^,^^13^ Long-term responders should undergo ongoing surveillance, ideally incorporating circulating tumor DNA to detect emergent resistant clones. Combination strategies with immune checkpoint blockade or ATR inhibitors may help address future resistance.

### 3.7 Practical lessons

This case highlights three clinically actionable principles: 1 Comprehensive genomic profiling should be initiated early to guide precision therapy.

2 While Prostate Cancer Clinical Trials Working Group 3 allows mCRPC diagnosis using PSA and CT, baseline and periodic bone scintigraphy remain desirable when feasible.
3 Olaparib monotherapy can produce exceptional, durable, and well-tolerated disease control in selected BRCA2-mutated patients, underscoring the value of continued documentation of long-term responders.

## 4 Conclusion

We report a BRCA2-mutated mCRPC patient who achieved a durable complete response to olaparib for 27 months without adverse events. Although bone scintigraphy was not performed at diagnosis, disease assessment was supported by PSA kinetics and CT, and later confirmed by a BSI of 0.00. This case highlights the importance of early genomic testing and continued monitoring.

## CRediT authorship contribution statement

**Fumihiro Ito:** Writing – original draft, Investigation, Formal analysis, Data curation, Conceptualization. **Koki Kobayashi:** Investigation. **Gaku Hayashi:** Investigation. **Shunsuke Kamijo:** Investigation. **Takashi Fujita:** Writing – review & editing, Supervision.

## Disclosure

The authors declare no conflicts of interest.

## Ethics approval

In accordance with institutional policy, ethics committee approval was not required for a single-patient case report that does not include identifiable personal information.

## Informed consent

Written informed consent for publication, including clinical data and images, was obtained from the patient and is available to the Editorial Office upon request.

## Study registration

Not applicable (single-patient case report; no trial registration).

## Funding

This research did not receive any specific grant from funding agencies in the public, commercial, or not-for-profit sectors.
